# MRI fat suppression techniques in traumatic and entrapment-related brachial plexus conditions: A scoping review^✰^

**DOI:** 10.1016/j.ejro.2026.100809

**Published:** 2026-08-15

**Authors:** Brian O. Molokwu, Rohan I. Suresh, Benjamin J. Park, Spencer C. Moore, Dhiraj R. Sibala, Alice Chu, Aleksandra M. McGrath, Pawel Szaro

**Affiliations:** a Department of Orthopaedics, Rutgers New Jersey Medical School, Newark, NJ, United States; b Department of Clinical Sciences, Umeå University, Umeå, Sweden; c Department of Diagnostics and Intervention (Orthopaedics), Umeå University, Umeå, Sweden; d Department of Radiology, Institute of Clinical Sciences, Sahlgrenska Academy, University of Gothenburg, Gothenburg, Sweden; e Department of Musculoskeletal Radiology, Sahlgrenska University Hospital, Gothenburg, Sweden; f Department of Clinical Anatomy, Medical University of Warsaw, Poland

**Keywords:** Brachial plexus injuries, Myelography, Magnetic resonance imaging, Fat suppression techniques, Diagnostic imaging

## Abstract

**Purpose:**

Traumatic and entrapment-related brachial plexus injuries are diagnostically complex conditions that often lead to substantial functional deficits. Magnetic resonance imaging (MRI) remains the modality of choice for preoperative evaluation, particularly when combined with fat-suppression techniques that enhance visualization of neural structures. Despite their role, the frequency and context of fat-suppression technique usage in brachial plexus imaging remain poorly defined.

**Methods:**

This scoping review aimed to systematically identify and categorize fat-suppression methods reported in MRI protocols for traumatic and entrapment-related brachial plexus injuries. A search of PubMed, Cochrane, CINAHL, and Web of Science was performed from inception to October 2023, following the Arksey and O’Malley methodological framework and reported in accordance with PRISMA-ScR guidelines. Studies were eligible if they described MRI protocols for mechanical brachial plexus injuries; non-mechanical etiologies (e.g., inflammatory or neoplastic conditions) were excluded.

**Results:**

Of 8647 screened records, 98 studies met inclusion criteria. Short tau inversion recovery (STIR) was the most frequently reported 2D fat suppression method (n = 37). Among 3D sequences, 3D STIR with sampling perfection with application-optimized contrasts using different flip-angle evolutions (SPACE) was most commonly reported. Most protocols utilized 1.5 Tesla MRI systems, a substantial minority employed 3 T systems. Dixon techniques were noted with increasing frequency in more recent literature.

**Conclusion:**

Our findings demonstrate sustained reporting of STIR while highlighting the increasing use of Dixon and advanced 3D sequences in recent publications. These results provide a comprehensive reference point for radiologists, suggesting areas for protocol optimization and standardization in brachial plexus pathology.

## Highlights

• STIR was the most frequently reported 2D fat-suppression technique.
• 3D STIR SPACE was the most frequently reported 3D sequence.
• Dixon appeared increasingly in more recent publications.
• Most included studies reported using 1.5-T MRI systems.
• Inconsistent protocol reporting limits comparison and standardization.

## 1 Introduction

Traumatic and entrapment injuries to the brachial plexus vary in severity and occur when the brachial plexus is stretched, compressed, or avulsed. These injuries affect a range of age groups, from neonates with neonatal brachial plexus palsy to adults with traumatic injuries and entrapment conditions often resulting in pain, numbness, and weakness. Recovery is challenging due to multiple factors such as the intricate physiology of nerve regeneration, or the presence of pre-surgical delay [1], [2]. A primary reason for delayed surgical intervention is the difficulty in diagnosing this injury [1], [2], [3]. Additionally, both traumatic brachial plexus injuries and entrapment of brachial plexus are relatively rare, potentially limiting clinician experience [3], [4]. In the current literature, Magnetic resonance imaging (MRI) is the most widely accepted imaging tool for the diagnosis of traumatic and entrapment related brachial plexus injuries [5], [6], [7]. Some studies have shown it has superior diagnostic capabilities compared to nerve and muscle electrophysiology studies as well as ultrasound [8], [9], [10], [11]. MRI is useful not only for diagnosing brachial plexopathies but also aids in surgical planning. Accurate identification of the location and extent of brachial plexus injury is important for selecting the appropriate treatment and planning surgical reconstruction.

MRI requires careful selection of sequences to accurately visualize brachial plexus pathology, with fat suppression techniques playing a key role [12]. These techniques improve contrast by reducing fat signals, making nerves and injuries clearer for diagnosis. Chemical Shift Selective Fat Saturation (CHESS/Fat-Sat) and Short Tau Inversion Recovery (STIR) were the first fat suppression techniques described for imaging nerves, improving nerve-to-background contrast [12]. However, newer techniques offering potential technical advantages are constantly being developed, such as the Dixon technique which offers effective fat-water separation capabilities, increased robustness to field inhomogeneities/susceptibility artifacts, and increased flexibility for different contrasts [13], [14]. The reported use in the literature of these newer, advanced fat suppression techniques remains unexplored.

Within the broader scope of MRI, specialized imaging modalities have been developed to further enhance nerve visualization. Magnetic Resonance Neurography (MRN) is a valuable non-invasive tool for assessing peripheral nerves, particularly for entrapments and injuries. It employs high-resolution T2-weighted sequences against a fat-suppressed background. A prime example is the 3D turbo spin echo with Sampling Perfection with Application optimized Contrast using different flip angle Evolution (SPACE) STIR sequence, which optimizes fat suppression and enables multi-plane imaging [12]. In heavily T2-weighted 3D MR neurography, intravenous gadolinium may improve vascular suppression through T2 shortening, thereby increasing nerve conspicuity; however, its use is sequence- and indication-dependent [15], [16]. Although advanced fat suppression techniques show promise for diagnosing brachial plexus injuries, studies on their reported use in the literature also remain limited, much like the Dixon technique. A broader lack of comprehensive research exists regarding the most commonly used fat suppression methods and the clinical adoption of emerging techniques. Familiarity with the adoption patterns, strengths, weaknesses of these methods can help guide protocol design, promote standardization, and by extension - improve diagnostic accuracy. Identifying utilization trends may also provide insight into the integration of newer techniques into clinical practice [17].

This scoping review aims to examine the existing literature to assess which MRI fat suppression and advanced techniques are used in diagnosing traumatic and entrapment-related brachial plexus injuries (TEBPI) in both adult and pediatric populations and to explore the reported use in the literature of emerging fat suppression techniques.

## 2 Materials and methods

This scoping review applied Arksey and O′Malley’s framework for scoping reviews, as modified by Levac et al. [18], [19] (see Supplementary Material 1). We adhered to the Preferred Reporting Items for Systematic reviews and Meta-Analyses Extension for Scoping Reviews (PRISMA-ScR) guidelines [20].

### 2.1 Search strategy

The search strategy was developed through discussion amongst the authors and consultation with an information specialist (university librarian). A search of PubMed, Cochrane, Cumulative Index to Nursing and Allied Health Literature (CINAHL) and Web of Science were searched from database inception through 10/05/2023. Boolean searches were performed with terms appertaining to “Brachial Plexus Injury” and “Magnetic Resonance Imaging”. No time limits were applied, and databases were searched for articles in English that focused on human subjects from inception to the date the search was performed (10/5/2023). These databases only host published articles, and do not contain any protected health information. Therefore, this study was exempt from IRB approval.

### 2.2 Study selection

After the database searches were completed, duplicates were removed and the remaining articles were independently screened by five reviewers (B.M., R.S., B.P., S.M., and D.R.S.). The review team performed the title, abstract and full-text level selection according to the inclusion and exclusion criteria listed below. All disagreements within the review team were resolved by three of the senior authors (A.C., A.M. and PS). Only original articles were included in this study. Inclusion criteria involved studies describing MRI protocols for the diagnosis of TEBPI. To identify these conditions, the studies had to include brachial plexus conditions caused by an external injury or entrapment that did not involve organic causes such as inflammation or tumors. MRI findings in inflammatory brachial plexus neuropathy are typically absent or discrete. Tumors on the other hand, were excluded because MRI protocols for tumor evaluation differ significantly from those for trauma. Tumor protocols prioritize contrast-enhanced sequences to visualize tumor morphology and its relationship to the brachial plexus, whereas trauma protocols emphasize MR neurography and typically do not involve contrast; this review aims to evaluate sequences specifically optimized for traumatic indications. Thus, the exclusion criteria included studies that do not focus on mechanical brachial injuries such as trauma or entrapment, or studies that do not use MRI as an imaging modality. These excluded studies were categorized as either “wrong study design”, “wrong intervention”, “wrong indication”, or “wrong patient population” in our selection process. Studies that included MRI in addition to other imaging modalities such as computed tomography (CT) or ultrasound were included. Studies concentrating on MRI imaging of the sequelae of brachial plexus injury, as well as those involving cadaveric models, were excluded. For example, one article that discussed neonatal brachial plexus injury but primarily focused on glenohumeral dysplasia was ultimately excluded during our selection process. Additionally, papers that were reviews, case reports, textbooks, letters to the editors, or written in a non-English language were also excluded from the study.

### 2.3 Data extraction

Data extraction strategy was developed by P.S. in consultation with the other authors. Extraction was performed similarly to study selection with two reviewers collecting data independently and any disagreements resolved in consultation with P.S. Data extraction was performed by B.M., R.S. and D.R.S. Data included publication date, magnetic field strength, patient age, two-dimensional (2D) fat-suppression techniques, three-dimensional (3D) MRI sequences used for brachial plexus imaging, and advanced MRI techniques. The 2D fat-suppression techniques included Dixon, short tau inversion recovery (STIR), chemical shift selective fat saturation (CHESS/Fat-Sat), spectral-spatial resonance frequency (SSRF), principle of selective excitation technique (Pro-SET), water excitation technique (WET), spectral presaturation with inversion recovery (SPIR), and spectral adiabatic inversion recovery (SPAIR). The 3D sequences included STIR with Sampling Perfection with Application-optimized Contrasts using different flip-angle Evolution (STIR SPACE), T2-weighted STIR SPACE, 3D STIR, 3D T2-weighted STIR, and Fast Imaging Employing Steady-state Acquisition (FIESTA). A complete extraction table summarizing these variables across all included studies is available in Supplementary Material 2. Although not fat suppression techniques themselves, these advanced imaging methods frequently incorporate fat suppression to enhance neural contrast and mitigate artifacts. Given their expanding role in brachial plexus imaging, we opted to extract data on these techniques to assess their utilization in conjunction with fat suppression and their integration into clinical and research workflows.

## 3 Results

**Fig. 1:**
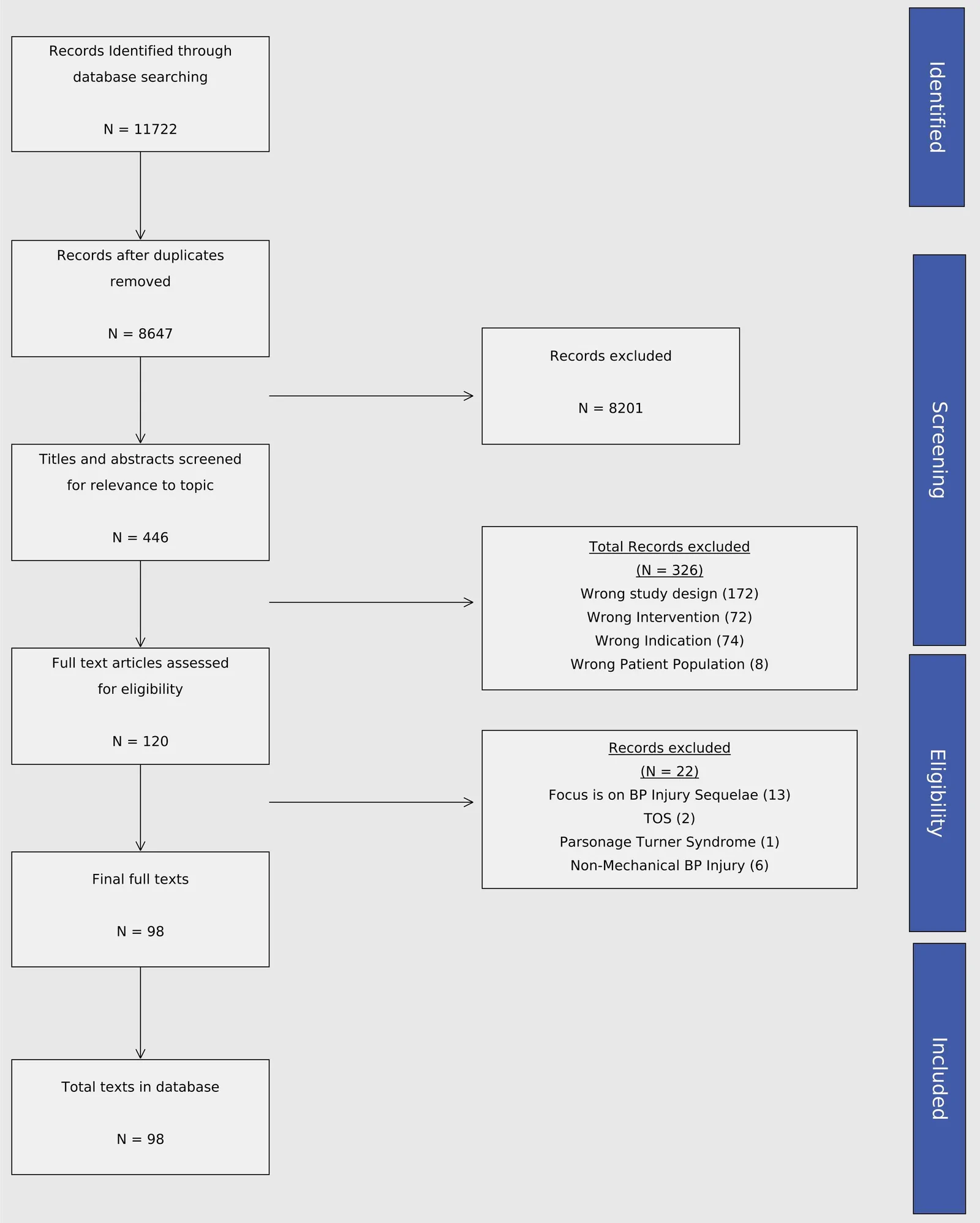
**PRISMA flow chart.** Flow chart outlining the various stages of the study selection process, including the identification, screening, eligibility assessment, and final inclusion of studies. TOS = Thoracic Outlet Syndrome.

After eliminating duplicates, a total of 8647 articles underwent screening based on their titles and abstracts, with each entry evaluated independently by two reviewers. This process led to the identification of 446 titles/abstracts relevant to the topic of interest. A subsequent screening of titles/abstracts with the appropriate indication (TEBPI) and intervention (MRI) generated 120 full text articles eligible for review. An assessment of these full texts was then performed, along with cross-referencing of citations, and a final round of exclusion criteria focusing on studies lacking detailed MRI protocols. During this stage, 13 studies focusing on brachial plexus injury sequelae, 6 on non-mechanical injuries, 2 on thoracic outlet syndrome, and 1 on Parsonage-Turner syndrome were excluded. Ultimately, 98 full text studies were deemed suitable for inclusion in the database. (Fig. 1). These selected studies spanned publication dates from 1993 to 2023. No formal bias assessment was conducted in keeping with scoping review methodology, and the MRI protocol parameters for diagnosing brachial plexus injury were summarized descriptively [17]. The final cohort of studies included can be viewed in Table 1. Further details on extracted imaging parameters for each study are provided in Supplementary Material 2.

**Table 1:** Characteristics of studies included in the final analysis.

Title of Study	Author	Year of Publication	Type of Imaging	Study Design	Reference Standard	Sample size	Clinical Setting
Magnetic Resonance Imaging of Obstetrical Brachial Plexus Injuries.	Abbott et al.	2004	MRI	Retro	Surgical exploration	15	Hospital
Advanced Radiological Work-up As an Adjunct to Decision in Early Reconstructive Surgery in Brachial Plexus Injuries	Abul-Kasim et al.	2010	MRI, CTM	Retro	Surgical exploration	7	Hospital
Diagnostic accuracy of MRI for traumatic adult brachial plexus injury: A comparison study with surgical findings	Acharya et al.	2020	MRI	Pro	Surgical exploration	35	Hospital
Do Not Forget the Brachial Plexus-prevalence Of Distal Brachial Plexus Pathology on Routine Shoulder MRI.	Antil et al.	2021	MRI	Retro	N/A	701	Hospital
Sensory Neuronopathy Involves the Spinal Cord and Brachial Plexus: A Quantitative Study Employing Multiple-echo Data Image Combination (MEDIC) And Turbo Inversion Recovery Magnitude (TIRM).	Bao et al.	2013	MRI	Pro	N/A	45	Hospital
Neonatal Magnetic Resonance Imaging Without Sedation Correlates with Injury Severity in Brachial Plexus Birth Palsy.	Bauer et al.	2017	MRI	Pro	N/A	9	Hospital
Two Clinical Tests Assessing Long Thoracic Nerve Function to Determine C5 And C6 Root Graft Eligibility in Patients with Brachial Plexus Injury	Bertelli et al.	2022	MRI	Pro	Surgical exploration	41	Hospital
Evaluation Of Mr-neurography In Diagnosis and Treatment in Peripheral Nerve Surgery of the Upper Extremity: A Matched Cohort Study.	Boecker et al.	2022	MRN	Retro	ENG, neurosonography	29	Hospital
Diagnostic Accuracy of Imaging Studies for Diagnosing Root Avulsions in Post-traumatic Upper Brachial Plexus Traction Injuries in Adults.	Bordalo-Rodrigues et al.	2020	MRI, CTM	Pro	Surgical exploration	52	Hospital
Brachial Plexus Injuries: Diagnosis Performance and Reliability of Everyday Tools.	Caporrino et al.	2014	MRI	Retro	Surgical exploration	102	Hospital
Diagnosis Of Root Avulsions in Traumatic Brachial Plexus Injuries: Value of Computerized Tomography Myelography and Magnetic Resonance Imaging.	Carvalho et al.	1997	MRI, CTM	Pro	Surgical exploration	135	Hospital
Value Of Clinical Findings, Electrodiagnosis and Magnetic Resonance Imaging in the Diagnosis of Root Lesions in Traumatic Brachial Plexus Injuries.	Chanlalit et al.	2005	MRI, EMG	Retro	Surgical exploration	175	Hospital
Differential Diagnosis Between Pre- and Postganglionic Adult Traumatic Brachial Plexus Lesions by Ultrasonography.	Chen et al.	2011	MRI, US	Pro	Surgical exploration	40	Hospital
Value Of Enhancement Technique In 3D-T2-STIR Images of the Brachial Plexus.	Chen et al.	2014	MRI	Pro	N/A	30	Hospital
Impact Of High Resolution 3 Tesla MR Neurography (MRN) On Diagnostic Thinking and Therapeutic Patient Management	Chhabra et al.	2016	MRN	Pro	N/A	81	Hospital
The Anatomy of the Brachial Plexus as Displayed by Magnetic Resonance Imaging: Technique and Application.	Collins et al.	1995	MRI	Pro	Surgical exploration	235	Hospital
Compromising Abnormalities of the Brachial Plexus as Displayed by Magnetic Resonance Imaging.	Collins et al.	1995	MRI	Pro	N/A	175	Hospital
Accuracy Of MR Neurography In the Diagnosis of Brachial Plexopathy.	Crim et al.	2017	MRN	Retro	ENG, neurosonography	43	Hospital
Diagnostic Contribution of Contrast-enhanced 3D MR Imaging of Peripheral Nerve Pathology.	Deshmukh et al.	2021	MRI	Retro	N/A	60	Hospital
MRI Of the Brachial Plexus: A Review Of 51 Cases.	deVerdier et al.	1993	MRI	Retro	Surgical exploration	51	Hospital
Diagnostic Accuracy of Magnetic Resonance Imaging With 3-dimensional T2-SPACE Techniques for Preganglionic Injury of the Brachial Plexus	Doi et al.	2022	MRI	Retro	Surgical Exploration	119	Hospital
Cervical Nerve Root Avulsion in Brachial Plexus Injuries: Magnetic Resonance Imaging Classification and Comparison with Myelography and Computerized Tomography Myelography.	Doi et al.	2002	MRI	Retro	CT Myelography, Myelography	35	Hospital
Magnetic Resonance Neurography for the Evaluation of Peripheral Nerve, Brachial Plexus, And Nerve Root Disorders.	Du et al.	2010	MRN	Retro	ENG, NCS	191	Hospital
The Value of Preoperative Examination and MRI For the Diagnosis of Graftable Roots in Total Brachial Plexus Palsy.	Echalier et al.	2019	MRI	Retro	Surgical exploration	27	Hospital
Role Of FIESTA Combined with Conventional MRI In the Evaluation of Traumatic Brachial Plexus Roots Injury	ElMogy et al.	2011	MRI	Pro	Surgical exploration	16	Hospital
Axial T2-DRIVE MRI Myelography Is Highly Accurate in Diagnosing Preganglionic Traumatic Brachial Plexus Injuries: Why Pseudomeningoceles Should Not Be Used as a Primary Diagnostic Sign.	Elsakka et al.	2022	MRI	Pro	Surgical exploration	24	Hospital
Application Of Magnetic Resonance Neurography in the Evaluation of Patients with Peripheral Nerve Pathology	Filler et al.	1996	MRN	Pro	N/A	242	Hospital
Clinical Impact of Magnetic Resonance Neurography in Patients with Brachial Plexus Neuropathies.	Fisher et al.	2016	MRN	Retro	N/A	121	Hospital
Role Of MRI In the Diagnosis of Adult Traumatic and Obstetric Brachial Plexus Injury Compared to Intraoperative Findings	Gad et al.	2020	MRI	Pro	Surgical exploration	37	Hospital
Three-dimensional MR Myelography Of Traumatic Injuries of the Brachial Plexus.	Gasparotti et al.	1997	MRM	Pro	CT myelography, myelography	20	Hospital
Feasibility Of Diffusion Tensor Tractography of Brachial Plexus Injuries At 1.5 T.	Gasparotti et al.	2013	MRI, MRN	Pro	N/A	28	Hospital
Diagnostic Value and Surgical Implications of the Magnetic Resonance Imaging in the Management of Adult Patients with Brachial Plexus Pathologies.	Gerevini et al.	2008	MRI	Retro	N/A	115	Hospital
Assessment Of the Usefulness of X-ray Myelography and Magnetic Resonance Myelography, Performed with an Open Low-field Device, In Diagnosing Perinatal Preganglionic Injuries of the Brachial Plexus	Gosk et al.	2012	MRM	Retro	Xray myelography	40	Hospital
Clinical Significance of Cervical MRI In Brachial Plexus Birth Injury.	Grahn et al.	2019	MRI	Pro	N/A	157	Hospital
Evidence For Increased Magnetic Resonance Imaging Signal Intensity and Morphological Changes in the Brachial Plexus and Median Nerves of Patients with Chronic Arm and Neck Pain Following Whiplash Injury.	Greening et al.	2018	MRI	Cross sectional	Clinical examination	23	Physical therapy and orthopaedic clinics
Brachial Plexus Ultrasound and MRI In Children with Brachial Plexus Birth Injury.	Gunes et al.	2018	MRI, US	Pro	MRI	55	Hospital
Value Of Shoulder US Compared to MRI In Infants with Obstetric Brachial Plexus Paralysis.	Gunes et al.	2021	MRI, US	Pro	MRI	42	Hospital
The Application of Contrast Enhanced 3D-STIR-VISTA MR Imaging of the Brachial Plexus	Han et al.	2022	MRI	Pro	N/A	30	Hospital
Quantitative MR Neurography Of Brachial Plexus Lesions Based on Diffusivity Measurements	Hassan et al.	2018	MRN	Pro	N/A	34	Hospital
Brachial Plexus Traumatic Root Injury in Adults: Role of Different Non-Contrast MRI Sequences in Pre-Operative Assessment	Hassan et al.	2017	MRI	Pro	N/A	20	Hospital
The Role of Magnetic Resonance Imaging in the Management of Traction Injuries to the Adult Brachial Plexus.	Hems et al.	1999	MRI	Pro	surgical exploration	26	Hospital
Somatotopic Fascicular Lesions of the Brachial Plexus Demonstrated by High-resolution Magnetic Resonance Neurography.	Hilgenfeld et al.	2017	MRN	Pro	EMG	36	Hospital
Cervical Root Avulsion - Magnetic Resonance Imaging Findings and Comparison of Diagnostic Accuracy Between Magnetic Resonance Imaging and Conventional Myelography	Hong et al.	1996	MRI	Retro	CT myelography	35	Hospital
Diagnostic Function Of 3-tesla Magnetic Resonance Imaging for the Assessment of Brachial Plexus Injury	Hung et al.	2020	MRI	Retro	surgical exploration	60	Hospital
Magnetic Resonance Tractography of the Brachial Plexus: Step-by-step	Ibrahim et al.	2022	MRI	Pro	N/A	31	Hospital
Degree Of Agreement Between Electrodiagnostic Testing and Magnetic Resonance Imaging in the Evaluation of Brachial Plexopathy.	Kang et al.	2019	MRI	Retro	Electrodiagnostic study	69	Hospital
Improved Brachial Plexus Visualization Using an Adiabatic Imsde-prepared STIR 3D TSE.	Klupp et al.	2019	MRN	Pro	N/A	22	Hospital
Correlation Of Preoperative MRI With the Long-term Outcomes of Dorsal Root Entry Zone Lesioning for Brachial Plexus Avulsion Pain.	Ko et al.	2016	MRI	Retro	N/A	15	Hospital
Diagnostic Performance of Diffusion-weighted MR Neurography As an Adjunct to Conventional MRI For the Assessment of Brachial Plexus Pathology.	Kwee et al.	2022	MRN	Pro	MRI	60	Hospital
Value Of High-resolution MRI In the Diagnosis of Brachial Plexus Injury in Infants and Young Children	Lao et al.	2022	MRI	Retro	EMG	26	Hospital
Traumatic Brachial Plexus Injury: A Study Of 510 Surgical Cases from Multicenter Services in Guangxi, China.	Li et al.	2019	MRI	Pro	CT	510	Hospital
MRI Neurography Findings in Patients with Idiopathic Brachial Plexopathy: Correlations with Clinical-neurophysiological Data in Eight Consecutive Cases.	Luigetti et al.	2013	MRN	Pro	N/A	8	Hospital
Additive Value of Magnetic Resonance Neurography in Diagnosis of Brachial Plexopathy: A Cross-section Descriptive Study	Mabrouk et al.	2021	MRN	Retro	Clinical examination	40	Hospital
Magnetic Resonance Imaging in Traumatic Brachial Plexopathy: A Guiding Light for Surgeons	Manzoor et al.	2021	MRI	Pro	Surgical exploration	40	Hospital
Diagnostic Performance of MRI And MR Myelography In Infants with a Brachial Plexus Birth Injury.	Medina et al.	2006	MRI, MRM	Pro	Surgical exploration, MEP, SEP	31	Hospital
Surgical Treatment of Brachial Plexus Injuries.	Mehta et al.	1993	MRI	Pro	Surgical exploration	99	Hospital
Brachial Plexopathy in Infants After Traumatic Delivery: Evaluation with MR Imaging.	Miller et al.	1993	MRI	Pro	N/A	5	Hospital
Non-traumatic Brachial Plexopathies, Clinical, Radiological and Neurophysiological Findings from a Tertiary Centre.	Mullins et al.	2007	MRI	Retro	EMG, NCS	25	Hospital
Magnetic Resonance Neurography of the Brachial Plexus Using 3D SHINKEI: Comparative Evaluation with Conventional Magnetic Resonance Sequences for the Visualization of Anatomy and Detection of Nerve Injury At 1.5 t	Nair et al.	2021	MRN	Pro	N/A	24	Hospital
Magnetic Resonance Myelography in Brachial Plexus Injury.	Nakamura et al.	1997	MRN	Pro	CT myelography, myelography	10	Hospital
The Diagnostic Value of MRI In Traumatic Brachial Plexus Injury.	Ochi et al.	1994	MRI	Pro	EMG, Surgical exploration	34	Hospital
Correlation Of Magnetic Resonance Imaging (Neurography) And Electrodiagnostic Study Findings with Intraoperative Findings in Post Traumatic Brachial Plexus Palsy	Patel et al.	2022	MRN	Pro	Electrodiagnostic study	48	Hospital
Clinical, Electrophysiological, And Imaging Findings in Childhood Brachial Plexus Injury	Portwood et al.	2022	MRI	Retro	Electrodiagnostic study	21	Hospital
Diagnostic Value and Surgical Implications of the 3D DW-SSFP MRI On the Management of Patients with Brachial Plexus Injuries.	Qin et al.	2016	MRN	Pro	EMG, Surgical exploration, SEP	33	Hospital
Diagnostic Value of Magnetic Resonance Neurography in Cervical Radiculopathy: Plexus Patterns and Peripheral Nerve Lesions.	Schwarz et al.	2018	MRN	Pro	Electrodiagnostic study	24	Hospital
Non-sedated Rapid Volumetric Proton Density MRI Predicts Neonatal Brachial Plexus Birth Palsy Functional Outcome.	Shen et al.	2017	MRI	Pro	N/A	9	Hospital
Magnetic Resonance Neurography in Children with Birth-related Brachial Plexus Injury.	Smith et al.	2008	MRN	Pro	Electrodiagnostic study	11	Hospital
MRI Evaluation of Nerve Root Avulsion in Neonatal Brachial Plexus Palsy: Understanding the Presence of Isolated Dorsal/Ventral Rootlet Disruption.	Smith et al.	2021	MRI	Retro	Surgical exploration	60	Hospital
Assessment Of Obstetric Brachial Plexus Injury with Preoperative Ultrasound.	Smith et al.	2016	MRI, US	Retro	Surgical exploration	8	Hospital
Post-contrast 3D Inversion Recovery Magnetic Resonance Neurography for Evaluation of Branch Nerves of the Brachial Plexus.	Sneag et al.	2020	MRI	Pro	N/A	18	Hospital
Prospective Respiratory Triggering Improves High-resolution Brachial Plexus MRI Quality.	Sneag et al.	2019	MRI	Pro	N/A	25	Hospital
High-resolution MRI Evaluation of Neonatal Brachial Plexus Palsy: A Promising Alternative to Traditional CT Myelography.	Somashekar et al.	2014	MRI	Pro	Surgical exploration	13	Hospital
Usefulness Of IDEAL T2 Imaging for Homogeneous Fat Suppression and Reducing Susceptibility Artefacts in Brachial Plexus MRI At 3.0 T.	Tagliafico et al.	2016	MRI	Pro	N/A	80	Hospital
MR Imaging of the Brachial Plexus: Comparison Between 1.5-T And 3-T MR Imaging: Preliminary Experience.	Tagliafico et al.	2011	MRI	Pro	N/A	60	Hospital
Brachial Plexus Assessment with Three-dimensional Isotropic Resolution Fast Spin Echo MRI: Comparison with Conventional MRI At 3.0 T.	Tagliafico et al.	2012	MRI	Pro	N/A	14	Hospital
Diagnostic Accuracy of MRI In Adults with Suspect Brachial Plexus Lesions: A Multicentre Retrospective Study with Surgical Findings and Clinical Follow-up as Reference Standard.	Tagliafico et al.	2012	MRI	Pro	Surgical exploration	157	Hospital
Concordance And Discrepancy Between Electrodiagnosis and Magnetic Resonance Imaging in Cervical Root Avulsion Injuries.	Tsai et al.	2006	MRI	Pro	EMG	37	Hospital
The Diagnostic Value of CT Myelography, MR Myelography, And Both in Neonatal Brachial Plexus Palsy.	Tse et al.	2014	MRM	Retro	CT myelography	19	Hospital
MR Neurography In Traumatic Brachial Plexopathy.	Upadhyaya et al.	2015	MRN	Pro	Surgical exploration	20	Hospital
A Decade of Imaging Patients with Traumatic Brachial Plexopathy: What Have We Learned?	Upadhyaya et al.	2023	MRN	Pro	N/A	134	Hospital
MR Neurography In Traumatic, Non-obstetric Paediatric Brachial Plexopathy.	Upadhyaya et al.	2018	MRN	Pro	N/A	25	Hospital
Diagnosis Of Nerve Root Avulsion Injuries in Adults with Traumatic Brachial Plexopathies: MRI Compared with CT Myelography	Van der Linde et al.	2015	MRI, MRM	Retro	NA	16	Hospital
Detection Of Root Avulsion in the Dominant C7 Obstetric Brachial Plexus Lesion: Experience with Three-dimensional Constructive Interference in Steady-state Magnetic Resonance Imaging and Electrophysiology.	Van Ouwerkerk et al.	2005	MRI	Pro	Surgical Exploration	10	Hospital
Use Of Magnetic Resonance Imaging to Diagnose Brachial Plexus Injuries	Veronesi et al.	2018	MRI	Pro	Surgical Exploration	3	Hospital
The Diagnostic Accuracy Of 1.5 T Magnetic Resonance Imaging for Detecting Root Avulsions in Traumatic Adult Brachial Plexus Injuries.	Wade et al.	2018	MRI	Retro	Surgical Exploration	29	Hospital
Diffusion Tensor Imaging for Diagnosing Root Avulsions in Traumatic Adult Brachial Plexus Injuries: A Proof-of-concept Study	Wade et al.	2020	MRI	Cross sectional	N/A	19	Hospital
Periscalene Soft Tissue: The New Imaging Hallmark in Erb Palsy.	Wandler et al.	2010	MRI	Retro	N/A	37	Hospital
The Application of Paramagnetic Contrast-based T2 Effect To 3D Heavily T2W High-resolution MR Imaging of the Brachial Plexus and Its Branches.	Wang et al.	2016	MRN	Pro	N/A	30	Hospital
Modified Pathological Classification of Brachial Plexus Root Injury and Its MR Imaging Characteristics.	Yang et al.	2014	MRI	Retro	N/A	86	Hospital
Predicting Healthy C5 Spinal Nerve Stumps Eligible for Grafting With MRI, Tinel Test, And Rhomboid Electromyography: A Retrospective Study Of 295 Consecutive Brachial Plexus Surgeries.	Yeow et al.	2021	MRI	Retro	EMG	251	Hospital
Clinical Assessment, MRI, And EMG In Congenital Brachial Plexus Palsy.	Yilmaz et al.	1999	MRI	Pro	EMG	13	Hospital
A Robust 3D Fast Spin-echo Technique for Fast Examination of the Brachial Plexus	Yoon et al.	2022	MRI	Cross sectional	N/A	14	Hospital
Three-tesla Magnetic Resonance Neurography of the Brachial Plexus in Cervical Radiculopathy.	Yoshida et al.	2015	MRN	Retro	N/A	12	Hospital
Clinical Value and Diagnostic Accuracy Of 3.0 T Multi-parameter Magnetic Resonance Imaging in Traumatic Brachial Plexus Injury.	Zhang et al.	2018	MRI	Pro	EMG, Surgical exploration	53	Hospital
The Effects of Three Different Contrast Agents (Gd-bopta, Gd-dtpa, And Gd-dota) On Brachial Plexus Magnetic Resonance Imaging	Zhang et al.	2021	MRI	Pro	N/A	60	Hospital
Segmented Echo Planar MR Imaging of the Brachial Plexus with Inversion Recovery Magnetization Preparation At 3.0 t.	Zhang et al.	2008	MRI	Pro	N/A	50	Hospital
Mri-based Optimization Design of the Pre-spinal Route of Contralateral C7 Nerve Transfer for Spastic Arm Paralysis	Zhao et al.	2022	MRI	Pro	N/A	80	Hospital
Increased Diagnostic Accuracy of Post-contrast MR 3D-STIR For Brachial Plexus Injury	Chen et al.	2016	MRI	Pro	N/A	48	Hospital

Abbreviations: MRI, magnetic resonance imaging; CT, computed tomography; CTM, computed tomography myelography; MRN, magnetic resonance neurography; MRM, magnetic resonance myelography; US, ultrasound; BP, brachial plexus; Pro, prospective; Retro, retrospective; ENG, electroneurography; EMG, electromyography; MEP, motor evoked potentials; SEP, somatosensory evoked potentials; NCS, nerve conduction study; N/A, not applicable.

**Fig. 2:**
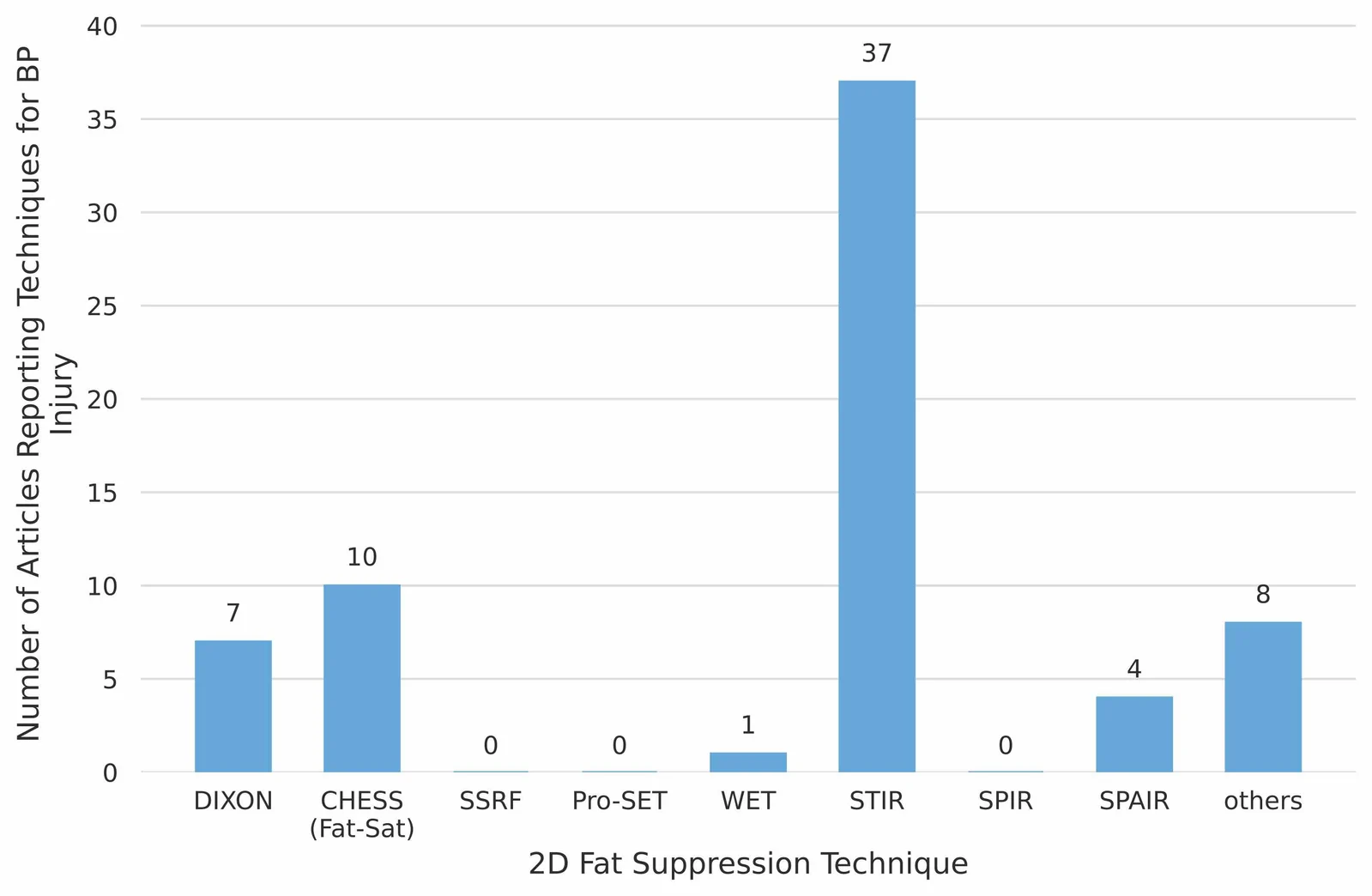
**Bar graph illustrating distribution of articles reporting various 2-dimensional fat suppression techniques for brachial plexus injury.** STIR = Short Tau inversion Recovery, CHESS = Chemical Shift Selective fat saturarion, SPAIR = Spectral Adiabatic Inversion Recovery, SSRF = Spectral-Spatial Resonance Frequency, Pro-SET = Principle of Selective Excitation, WET = Water Excitation Technique, SPIR = Spectral Presaturation with Inversion Recovery.

When considering the magnetic field strength used, a majority of studies utilized 1.5 T systems (n = 53), with 3 T being the second most reported magnetic field strength (n = 39). Low-strength systems such as 1 T and 0.5 T were each reported twice, while 0.23 T was reported once [21], [22], [23], [24]. None of the authors reported the use of a higher field system such as 7 T scanners. Over half of the studies reported the use of fat suppression for 2D sequences (n = 52), with STIR being the predominant choice (n = 37), while CHESS (Fat Saturation) was utilized in ten studies, and Dixon in seven studies, SPAIR in four. WET was used in one study (Fig. 2). [25], [26]. The reported 2D fat suppression techniques and 3D MRI sequences, categorized by magnetic field strength, are summarized in Table 2.

**Table 2:** Reported 2D fat suppression techniques and 3D MRI sequences used in brachial plexus imaging, categorized by magnetic field strength.

**Category**	**Technique**	**Total Papers (N)**	**Papers Reporting 1.5 T (N)**	**Papers Reporting 3 T (N)**
2D fat suppression	STIR	37	23	15
	CHESS	10	6	6
	Dixon	7	4	4
	SPAIR	4	1	3
	Others	7	5	3
3D MRI sequence	STIR SPACE	8	5	4
	T2 STIR SPACE	6	2	4
	STIR	4	4	2
	T2 STIR	2	0	2
	FIESTA	6	6	0
	Others	8	3	5

**Abbreviations:** STIR, short tau inversion recovery; CHESS, chemical shift selective fat saturation; SPAIR, spectral adiabatic inversion recovery; SPACE, Sampling Perfection with Application-optimized Contrasts using different flip-angle Evolutions; FIESTA, fast imaging employing steady-state acquisition.

Field-strength categories were not mutually exclusive because some studies reported examinations performed at more than one magnetic field strength; therefore, the field-strength columns may sum to more than the total number of studies for a technique.

**Fig. 3:**
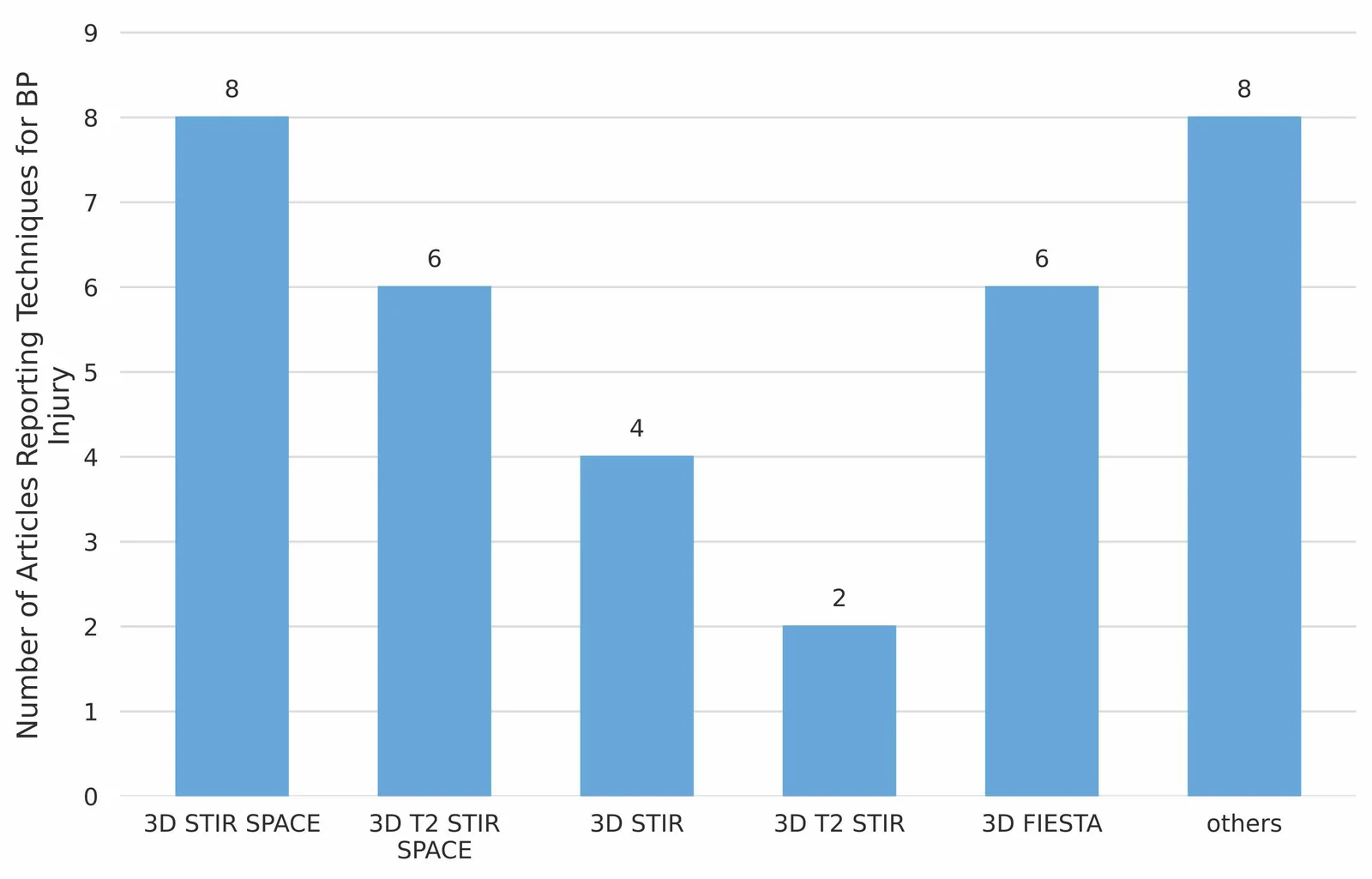
**Bar graph illustrating the distribution of articles reporting various 3-dimensional MRI sequences used in brachial plexus imaging.** STIR = short tau inversion recovery; SPACE = Sampling Perfection with Application-optimized Contrasts using different flip-angle Evolutions; FIESTA = fast imaging employing steady-state acquisition; TSE = turbo spin echo; IMSDE = improved motion-sensitized driven equilibrium; DW = diffusion-weighted; SSFP = steady-state free precession.

**Fig. 4:**
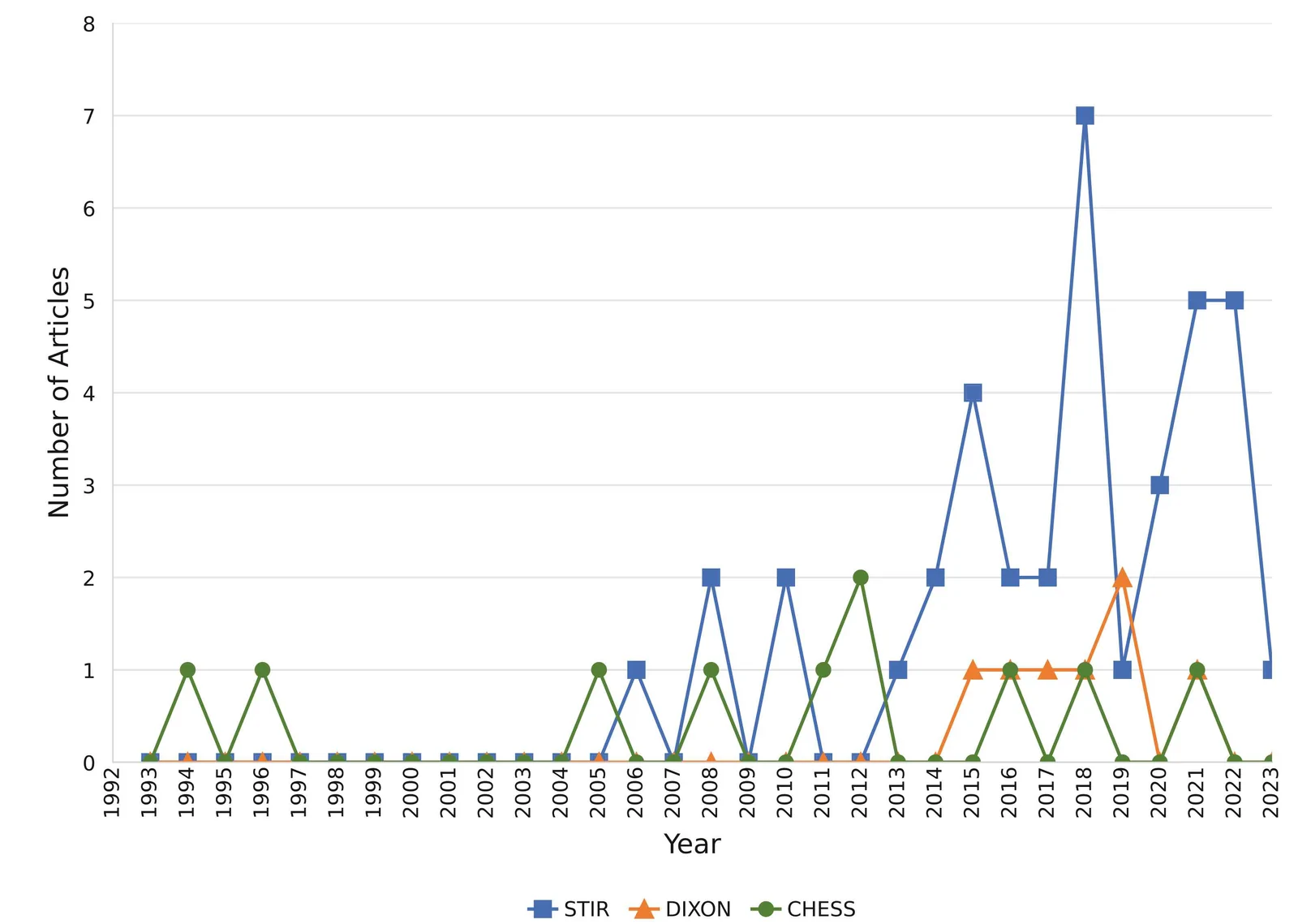
**Line graph illustrating the number of studies using STIR, Dixon and CHESS by publication year.** STIR = Short Tau inversion Recovery, CHESS = Chemical Shift Selective.

Thirty-four papers documented the utilization of 3D techniques. Among these, 3D SPACE STIR was the most reported sequence (N = 8); 3D FIESTA (N = 6) and 3D T2 STIR SPACE (N = 6) were the second most reported sequences; 3D STIR ( N = 4), 3D T2 STIR (N = 2), 3D T2 SPACE (N = 2), 3D-T2 STIR with 3D TSE + SPACE (N = 1), 3D IMSDE STIR + TSE (N = 1), 3D Nerve SHINKEI (N = 1), 3D DW SSFP (N = 1), Triple echo Dixon (N = 1), and CUBE STIR (N = 1) (Fig. 3). The oldest study in our subgroup that reported use of Dixon occurred in 2015, while the oldest study that reported use of STIR dated back to 2005 (Fig. 4). The oldest study that reported CHESS/Fat-Sat dates back even earlier to 1994.

While use of advanced MRI techniques such as diffusion weighted imaging (DWI) and diffusion tensor imaging (DTI) varied across studies, most of the studies that included these techniques specifically utilized MRI Myelography (N = 13), with DWI being reported in 12 studies, and DTI being reported in 3 studies.

## 4 Discussion

Our scoping review highlights the diverse use of fat suppression techniques in MRI for visualizing TEBPI and shows temporal trends in published fat suppression techniques over the last 30 years. We found that STIR was the most commonly reported 2D imaging technique, appearing in 37 studies, while among advanced 3D methods, 3D STIR SPACE was the most frequently utilized sequence. As MRI is a principal non-invasive imaging modality for evaluating these injuries, selecting the appropriate fat suppression technique remains critical for obtaining accurate diagnostic information [27], [28], [29]. Effective fat suppression may improve visualization of the affected nerve roots and the extent of injury, thereby supporting decisions regarding conservative versus surgical treatment and preoperative planning.

STIR, the most reported 2D technique, offers effective fat suppression while providing high contrast between nerve tissue and surrounding structures [30]. While comparative studies in the context of the brachial plexus are limited, Dixon has demonstrated robust and homogeneous fat–water separation in several anatomical regions [29]. We found that while novel methods such as Dixon are gaining acceptance due to their enhanced diagnostic capabilities, traditional techniques such as STIR remain prevalent.

The adoption of 3D MRI sequences used in brachial plexus imaging were also analyzed, with 3D STIR SPACE being the most frequently reported sequence (8 studies), followed by 3D T2-weighted STIR SPACE and 3D FIESTA. Even though 3D T2 STIR SPACE is known for its reliable nerve visualization due to varying T2 relaxation times, it was not the most reported. This may be attributed to inconsistent reporting of whether 3D sequences were acquired with T1- versus T2-weighted imaging. Isotropic 3D sequences enable multiplanar and curved-planar reconstructions, although spatial resolution, signal-to-noise ratio, and acquisition time vary according to the applied protocol. Nonetheless, 3D sequences offer enhanced through-plane resolution, which significantly improves the visualization of complex brachial plexus injuries, although their in-plane resolution is arguably inferior compared to high resolution 2D techniques [30], [31].

Next, our findings depict trends seen in the use of magnetic field strengths with various 2D and 3D techniques. Overall, amongst 2D techniques the 1.5 T was used more often than the 3 T magnetic field strength. The STIR technique showed a preference for using 1.5 T magnetic field strength over 3 T. The CHESS and Dixon techniques were equally divided between the two field strengths. The SPAIR technique, though less commonly reported, favored 3 T. Other techniques predominantly utilized 1.5 T, with fewer papers mentioning the use of 3 T. In contrast, the 3D techniques had similar reporting frequencies of 1.5 T and 3 T across all papers, except in the T2 STIR group, where both papers reported using 3 T, and in the FIESTA group, where all six papers reported using 1.5 T. Although 1.5 T remains widely used, 3 T has been shown to provide superior signal-to-noise and contrast-to-noise ratios. These benefits, however, are counterbalanced by higher specific absorption rate (SAR) limitations and greater susceptibility to magnetic-field inhomogeneity artifacts at 3 T [32]. Despite its documented advantages, the impact of 3 T versus 1.5 T on clinical outcomes for brachial plexus injuries remains uncertain [33].

Lastly, we assessed the use of advanced MRI techniques such as diffusion-weighted imaging (DWI) and diffusion tensor imaging (DTI) which provide insights into the microstructural organization of the brachial plexus. DTI enables nerve fiber tractography and the calculation of in-vivo biomarkers such as fractional anisotropy and mean diffusivity [34], [35]. Their limited reporting in our study likely reflects technical complexity, acquisition and post-processing requirements, and limited availability [36]. Further studies are needed to establish their diagnostic value in mechanical brachial plexus injuries. Variability in their application across studies highlights evolving technology and differing radiologist familiarity. Despite this, these methods hold promise for improving MRI diagnostic accuracy in mechanical brachial plexus injuries.

Benefits and drawbacks vary across fat suppression techniques. Fat saturation methods such as CHESS offer high signal-to-noise ratio (SNR) and relatively short acquisition times; however, they are sensitive to B0 inhomogeneity, especially in the brachial plexus region [17]**.** STIR, using a 180° RF pre-pulse, offers more reliable suppression but adds T1-weighting, which limits its use for tissues with similar T1 properties to fat. STIR has also been associated with poor signal-to-noise ratio and a susceptibility to pulsation artifacts [37]. SPIR and SPAIR enhance fat suppression through spectral pre-saturation; however, SPIR remains sensitive to B1 inhomogeneities, whereas SPAIR is generally less sensitive to B1 inhomogeneity than SPIR because it uses an adiabatic inversion pulse. Variations in utilization amongst various fat suppression techniques may be attributable to differences in diagnostic benefits versus limitations of these techniques relative to one another.

Our review findings align with and expand upon existing literature in this domain. STIR has been the most frequently reported 2D fat suppression technique in the literature for visualizing mechanical brachial plexus injuries, largely due to its longstanding status as the gold standard for fat suppression in MRI protocols [37], [38]. We hypothesize that radiologist familiarity has likely driven its continued and comparatively higher use over Dixon or CHESS [39]. The introduction of a new technique does not automatically lead to its clinical adoption. There is a tendency to rely on established methods until the efficacy of the new technique is sufficiently demonstrated through research. A significant barrier to the adoption of new techniques is the need for their implementation and optimization of existing machines, which demands both time and financial investment. However, as newer protocols offering superior visualization become more widely adopted, the reliance on STIR is likely to decrease in favor of these advanced fat suppression modalities. In the context of surgical planning for complex cases, STIR and Dixon both offer accentuated delineation of the neural plexus segments that are distinguishable from surrounding soft tissue. Our review suggests a shift towards the adoption of newer techniques. The increasing reporting of Dixon suggests growing interest in its technical advantages; however, direct comparative studies are needed to establish its diagnostic and clinical value relative to STIR [14], [40]. The prominence of 3D STIR SPACE in our review aligns with its reported advantages in the literature, particularly its ability to produce high-contrast images with effective fat suppression, making it ideal for assessing complex nerve injuries, such as in traumatic settings. Given the challenges in assessing the severity of postganglionic nerve damage (i.e. rupture versus contusion), 3D STIR SPACE’s ability to provide detailed imaging of both preganglionic and postganglionic segments of the brachial plexus makes it a valuable tool for comprehensive injury evaluation [41], [42], [43]. Furthermore, its high spatial resolution, its ability to minimize artifacts and capture subtle caliber changes are important for accurately assessing the intricate anatomy of the brachial plexus [44]. Additionally, the adaptability of STIR sequences across different MRI environments likely contributes to the widespread use of 3D STIR SPACE. Studies consistently highlight 3D STIR SPACE’s superior diagnostic accuracy in detecting nerve thickening, neuromas, and other pathological changes, explaining its frequent use in our review [28]. However, lack of flow compensation is a known limitation, particularly in detecting nerve root avulsions, where optimized acquisition planes or flow compensation may be required. While these adjustments improve visualization, they also extend scan time, potentially limiting clinical feasibility [43].

### 4.1 Limitations

Our study is not without its limitations. The variability in study designs may limit the generalizability of our findings. The included studies did not describe protocols and application areas in sufficient detail to differentiate between sequences used for diagnosis of traumatic brachial plexus injuries versus surgical planning. This is an important knowledge gap as an assessment of the volume of viable nerve tissue in proximal stump is of crucial importance for patients with more extensive injuries, especially viewed through the lens of trends in surgical techniques for treatment of TEBPI. Future studies should correlate MRI findings obtained with different fat suppression techniques with intraoperative findings to assess their diagnostic accuracy and value in surgical planning. Additionally, this review did not identify any studies utilizing high-field MRI systems (>3 T), limiting our ability to assess their potential advantages in brachial plexus imaging. Future research should explore whether these systems improve diagnostic accuracy or clinical outcomes. Our analysis of correlations between fat suppression techniques and magnetic field strengths does not account for the time of publication or regional variations in funding, both of which may influence the adoption of specific fat suppression techniques and MRI field strengths over time. Furthermore, many clinical protocols likely incorporate a combination of 2D and 3D imaging techniques rather than using them in isolation, which was not consistently specified in the included studies. Another limitation is the inherent subjectivity in scoping reviews, although we took measures to minimize reviewer bias by implementing standardized criteria for study selection and data extraction. Furthermore, variations in patient populations (e.g., pediatric vs. adult studies), and operator expertise could have influenced the reported findings. Lastly, the exclusion of non-English studies could have resulted in the omission of relevant data from other regions. Despite these limitations, the study provides valuable insights into current imaging practices and offers a comprehensive foundation that can guide future research and clinical decision-making.

### 4.2 Conclusion

A variety of fat suppression techniques are utilized in the imaging of traumatic and entrapment-related brachial plexus injuries, with STIR being the most frequently reported 2D technique. Dixon and advanced 3D sequences, particularly 3D STIR SPACE, appeared increasingly in more recent publications. Future research should compare these techniques using standardized protocols and clinically relevant reference standards, including intraoperative findings, to determine their diagnostic accuracy and value for treatment planning.

## Institutional review board

This study contains only de-identified data, and it does not contain any protected health information. Therefore, this study was exempt from IRB approval.

## Supplementary material

Supplementary Material 1 – PRISMA-ScR Checklist Completed checklist documenting adherence to the Preferred Reporting Items for Systematic Reviews and Meta-Analyses extension for Scoping Reviews (PRISMA-ScR) guidelines.

Supplementary Material 2 – Results of Individual Sources of Evidence Extracted data from all included studies, detailing MRI field strength, fat suppression techniques (2D and 3D), and use of advanced imaging sequences such as diffusion-weighted imaging, diffusion tensor imaging, and MRI myelography.

## CRediT authorship contribution statement

**McGrath Aleksandra:** Writing – review & editing, Supervision, Methodology, Conceptualization. **Pawel Szaro:** Writing – review & editing, Writing – original draft, Visualization, Supervision, Methodology, Conceptualization. **Alice Chu:** Validation, Supervision, Methodology, Conceptualization. **Moore Spencer:** Formal analysis, Data curation. **Sibala Dhiraj:** Methodology, Formal analysis, Data curation. **Suresh Rohan:** Writing – review & editing, Writing – original draft, Formal analysis, Data curation. **Park Benjamin:** Formal analysis, Data curation. **Molokwu Brian:** Writing – review & editing, Writing – original draft, Formal analysis, Data curation.

## Consent to participate

Not applicable

## Consent for publication

Not applicable.

## Ethics approval

Ethics approval was not required because this scoping review was based exclusively on previously published literature.

## Declaration of Generative AI and AI-assisted technologies in the writing process

During the preparation of this work, the authors used ChatGPT (OpenAI) to improve the language and readability of the manuscript. After using this tool, the authors reviewed and edited the content as needed and take full responsibility for the content of the publication.

## Data availability

The data supporting this systematic review are derived from publicly available literature sources. All relevant data are included in the article, and additional information can be accessed through the cited studies.

## Funding

This research did not receive any specific grant from funding agencies in the public, commercial, or not-for-profit sectors.

## Declaration of Competing Interest

The authors declare that they have no known competing financial interests or personal relationships that could have appeared to influence the work reported in this paper.

## Data Availability

All data extracted for this scoping review are provided in the article and its supplementary materials.

## Footnotes

Appendix A Supplementary data associated with this article can be found in the online version at doi:10.1016/j.ejro.2026.100809.
