# Supplementary material for: MRI fat suppression techniques in traumatic and entrapment-related brachial plexus conditions: A scoping review
Source: Eur J Radiol Open. 2026 Aug 15;17:100809. doi: 10.1016/j.ejro.2026.100809 (PMC13499448; doi:10.1016/j.ejro.2026.100809)
Supplement: Supplementary file 1 [file mmc1.pdf]

Results of Individual Sources of Evidence

author	Magnetic Field Strength (T)	Fat supression for 2D squences										Fat supression for 3D sequences		Advanced MRI techniques?			
		0= no 1=yes	DIXON	CHESS (Fa	SSRF	Pro-SET	WET	STIR	SPIR	SPAIR	others	Were performed? 1 yes, 0 no	What type of fat supression was used? - type the technique here	DWI	DTI	Long effective TE = MRI myelography	others
Abbott 2004	1.5	0	0	0	0	0	0	0	0	0	0	0	n/a	0	0	0	0
Abul-Kasim 2010	n/a	1	0	0	0	0	0	1	0	0	0	0	n/a	0	0	0	3D CISS
Acharya 2020	1.5	1	0	0	0	0	0	1	0	0	0	0	n/a	0	0	0	0
Antil 2021	3	1	0	1	0	0	0	0	0	0	0	0	n/a	0	0	0	0
Bao 2013	3	0	0	0	0	0	0	0	0	0	Satutration Band	0	n/a	0	0	0	MEDIC and TIRM
Bauer 2017	3	0	0	0	0	0	0	0	0	0	0	0	n/a	0	0	0	3D-PD MRI
Bertelli 2022	n/a	0	0	0	0	0	0	0	0	0	0	0	n/a	0	0	0	0
Boecker 2022	3	0	0	0	0	0	0	0	0	0	0	1	Fat spectral saturation	0	0	0	3d T2-w
Bordalo-Rodrigues 2020	1.5	1	0	0	0	0	0	1	0	0	0	1	FIESTA	0	0	0	0
Caporrino 2014	1.5	0	0	0	0	0	0	0	0	0	0	0	n/a	0	0	0	0
Carvalho 1997	1.5	0	0	0	0	0	0	0	0	0	0	0	n/a	0	0	0	0
Chanlalit 2005	1.5	1	0	1	0	0	0	0	0	0	0	0	n/a	0	0	0	0
Chen 2011	1.5	0	0	0	0	0	0	0	0	0	0	1	n/a	0	0	0	0
Chen 2014	3	1	0	0	0	0	0	1	0	0	0	1	3D-T2 STIR using 3D TSE(turbo spin echo) with SPACE	0	0	0	0
Chhabra 2016	3	1	0	0	0	0	1	0	0	1	IR-fs	0	n/a	1	0	0	0
Collins 1995	1.5	1	0	0	0	0	0	0	0	0	water bags	0	n/a	0	0	0	0
Collins 1995	1.5	1	0	0	0	0	0	0	0	0	water bags	0	n/a	0	0	0	0
Crim 2017	1.5 & 3	1	1	0	0	0	0	1	0	0	STIR-SPACE	1	3D-Stir	0	0	0	0
Deshmukh 2021	1.5 & 3	1	0	0	0	0	0	1	0	0	0	1	3D SPACE STIR and 3D STIR-FSE	0	0	0	0
deVerdier 1993	1.5	0	0	0	0	0	0	0	0	0	0	0	n/a	0	0	0	0
Doi 2022	1.5	1	0	0	0	0	0	1	0	0	0	1	3D-T2 SPACE	0	0	0	0
Doi 2002	1	0	0	0	0	0	0	0	0	0	0	0	n/a	0	0	0	0
Du 2010	1.5	1	0	0	0	0	0	1	0	0	0	0	n/a	0	0	0	3D-DESS
Echalier 2019	1.5	1	1	0	0	0	0	0	0	0	0	1	3D-Stir	0	0	0	0
ElMogy 2011	1.5	0	0	0	0	0	0	0	0	0	0	1	FIESTA	0	0	0	0
Elsakka 2022	1.5	0	0	0	0	0	0	0	0	0	0	1	3D-STIR	0	0	0	T2-DRIVE
Filler 1996	1.5	1	0	1	0	0	0	0	0	0	0	0	n/a	0	0	0	0
Fisher 2016	1.5	1	0	0	0	0	0	1	0	0	0	1	3D SPACE STIR; 3D-T2 SPACE; FIESTA	0	1	0	0
Gad 2020	1.5	0	0	0	0	0	0	0	0	0	0	1	3D T2 STIR SPACE	1	0	1	0
Gasparotti 1997	1.5	0	0	0	0	0	0	0	0	0	0	0	n/a	0	0	1	CISS
Gasparotti 2013	1.5	0	0	0	0	0	0	0	0	0	0	1	3D T2 STIR SPACE	0	1	1	0
Gerevini 2008	1.5	1	0	0	0	0	0	1	0	0	0	0	n/a	0	0	1	0
Gosk 2012	0.23	0	0	0	0	0	0	0	0	0	0	0	n/a	0	0	1	0
Grahn 2019	1.5	0	0	0	0	0	0	0	0	0	0	0	n/a	0	0	1	0
Greening 2018	1.5	1	0	0	0	0	0	1	0	0	0	0	n/a	0	0	0	0
Gunes 2018	1.5	1	0	0	0	0	0	1	0	0	0	0	n/a	1	0	0	CISS
Gunes 2021	1.5	0	0	0	0	0	0	0	0	0	0	0	n/a	0	0	0	TR/TE
Han 2022	3	1	0	0	0	0	0	1	0	0	0	0	n/a	1	0	0	STIR-VISTA
Hassan 2018	1.5	1	0	0	0	0	0	1	0	0	0	0	n/a	1	1	0	DWIBS
Hassan 2017	1.5	1	0	0	0	0	0	1	0	0	Scout GRE	1	FIESTA	0	0	1	3D FIESTA, Coronal STIR
Hems 1999	0.5	0	0	0	0	0	0	0	0	0	0	0	n/a	0	0	0	0
Hilgenfeld 2017	3	1	0	0	0	0	0	1	0	1	0	1	3D T2 SPACE STIR	0	0	0	SPACE STIR
Hong 1996	1	0	0	0	0	0	0	0	0	0	0	0	n/a	0	0	0	0
Hung 2020	3	1	0	0	0	0	0	1	0	0	0	1	3D T2 STIR	0	0	0	CISS
Ibrahim 2022	3	1	0	0	0	0	0	1	0	0	0	1	3D T2 SPACE STIR	0	0	0	SS-EPI (Single Shot echo-planar imaging sequence)
Kang 2019	3	0	0	0	0	0	0	0	0	0	0	0	n/a	0	0	0	0
Klupp 2019	3	1	0	0	0	0	0	1	0	0	0	1	3D IMSDE STIR / turbo Spin echo	0	0	0	IMSDE
Ko 2016	n/a	0	0	0	0	0	0	0	0	0	0	0	n/a	0	0	0	0
Kwee 2022	1.5 & 3	1	0	0	0	0	0	1	0	0	0	1	3D STIR	1	0	0	0
Lao 2022	3	0	0	0	0	0	0	0	0	0	0	1	3D STIR SPACE	0	0	0	0
Li 2019	n/a	0	0	0	0	0	0	0	0	0	0	0	n/a	0	0	0	0
Luigetti 2013	1.5	1	0	0	0	0	0	1	0	0	0	0	n/a	0	0	0	0
Mabrouk 2021	1.5	1	0	0	0	0	0	1	0	1	0	1	FIESTA	0	0	0	0
Manzoor 2021	1.5	1	0	0	0	0	0	1	0	0	0	0	n/a	0	0	0	CISS
Medina 2006	1.5	1	0	0	0	0	0	1	0	0	0	0	n/a	0	0	1	0
Mehta 1993	1.5	0	0	0	0	0	0	0	0	0	0	0	n/a	0	0	1	
Miller 1993	1.5	0	0	0	0	0	0	0	0	0	0	0	n/a	0	0	0	0
Mullins 2007	n/a	0	0	0	0	0	0	0	0	0	0	0	n/a	0	0	0	0
Nair 2021	1.5	1	0	0	0	0	0	1	0	0	0	1	3D nerve sheath signal increased with INKED rest tissue Rare Imaging (3D - SH	1	0	0	0
Nakamura 1997	1.5	0	0	0	0	0	0	0	0	0	0	0	n/a	0	0	1	0
Ochi 1994	0.1, 0.5 , 1.5	1	0	1	0	0	0	0	0	0	0	0	n/a	0	0	1	0
Patel 2022	3	1	0	0	0	0	0	1	0	0	0	0	n/a	1	0	0	0
Portwood 2022	n/a	0	0	0	0	0	0	0	0	0	0	0	n/a	0	0	0	0
Qin 2016	3	0	0	0	0	0	0	0	0	0	0	1	3D DW SSFP	1	0	0	3D PSIF
Schwarz 2018	3	1	0	1	0	0	0	1	0	1	0	0	n/a	0	0	0	0
Shen 2017	3	0	0	0	0	0	0	0	0	0	0	0	n/a	0	0	0	0
Smith 2008	1.5	1	0	1	0	0	0	0	0	0	0	0	n/a	0	0	0	0
Smith 2021	3	0	0	0	0	0	0	0	0	0	0	0	n/a	0	0	0	3D T2 DRIVE
Smith 2016	n/a	0	0	0	0	0	0	0	0	0	0	0	n/a	0	0	0	0
Sneag 2020	3	0	0	0	0	0	0	0	0	0	0	1	3D T2 STIR(post contrast vs pre contrast)	0	0	0	0
Sneag 2019	3	1	1	0	0	0	0	0	0	0	0	0	n/a	0	0	0	Proton Density?
Somashekar 2014	3	0	0	0	0	0	0	0	0	0	0	0	n/a	0	0	0	3D T2 DRIVE
Tagliafico 2016	3	1	1	1	0	0	0	0	0	0	0	0	n/a	0	0	0	0
Tagliafico 2011	1.5 & 3	1	0	1	0	0	0	0	0	0	0	0	n/a	0	0	0	0
Tagliafico 2012	3	1	0	1	0	0	0	0	0	0	0	0	n/a	0	0	0	3D FSE cube
Tagliafico 2012	1.5 & 3	1	0	1	0	0	0	0	0	0	0	0	n/a	0	0	0	0
Tsai 2006	1.5	0	0	0	0	0	0	0	0	0	0	1	FIESTA	0	0	1	0
Tse 2014	3	1	0	0	0	0	0	1	0	0	0	0	n/a	0	0	1	9
Upadhyaya 2015	1.5	1	1	0	0	0	0	1	0	0	0	1	SPACE STIR, SPACE	0	0	0	0
Upadhyaya 2023	1.5	1	0	0	0	0	0	1	0	0	SPACE	1	SPACE STIR, SPACE	0	0	0	0
Upadhyaya 2018	1.5	1	1	0	0	0	0	1	0	0	0	1	SPACE STIR, SPACE	0	0	0	0
vanderLinde 2015	1.5	0	0	0	0	0	0	0	0	0	0	0	n/a	0	0	0	0
vanOuwerkerk 2005	1.5	0	0	0	0	0	0	0	0	0	0	0	n/a	0	0	0	0
Veronesi 2018	1.5	0	0	0	0	0	0	0	0	0	0	0	n/a	1	0	0	0
Wade 2018	1.5	1	0	0	0	0	0	1	0	0	0	0	n/a	0	0	0	0
Wade 2020	3	0	0	0	0	0	0	0	0	0	0	0	n/a	0	0	0	0
Wandler 2010	1.5	0	0	0	0	0	0	0	0	0	0	0	n/a	0	0	0	0
Wang 2016	3	0	0	0	0	0	0	0	0	0	0	1	SPACE STIR	0	0	0	0
Yang 2014	3	0	0	0	0	0	0	0	0	0	0	0	n/a	0	0	0	0
Yeow 2021	1.5	1	0	0	0	0	0	1	0	0	0	0	n/a	1	0	0	0
Yilmaz 1999	1.5	0	0	0	0	0	0	0	0	0	0	0	n/a	0	0	0	0
Yoon	3	0	0	0	0	0	0	0	0	0	0	1	triple echo DIXON	0	0	0	0
Yoshida 2015	3	1	0	0	0	0	0	1	0	0	0	0	n/a	0	0	0	0
Zhang 2018	3	1	0	0	0	0	0	1	0	0	0	0	n/a	1	0	0	0
Zhang 2021	3	1	1	0	0	0	0	0	0	0	0	1	SPACE STIR	0	0	0	0
Zhang 2008	3	1	0	0	0	0	0	1	0	0	0	0	n/a	0	0	0	0
Zhao 2022	3	0	0	0	0	0	0	0	0	0	0	1	CUBE-STIR	0	0	0	0
Chen 2016	3	1	0	0	0	0	0	1	0	0	0	1	3D T2WI STIR and SPACE	0	0	0	0
